# Topical Chemotherapy for Ocular Surface Squamous Neoplasia: A Review of Adverse Effects and Their Clinical Management

**DOI:** 10.32604/or.2025.067221

**Published:** 2025-09-26

**Authors:** Lina Corgiolu, Giuseppe Giannaccare, Alberto Cuccu

**Affiliations:** Eye Clinic, Department of Surgical Sciences, University of Cagliari, Cagliari, 09124, Italy

**Keywords:** Ocular surface squamous neoplasia, topical chemotherapy, mitomycin C, 5-fluorouracil, interferon alpha-2b

## Abstract

Topical chemotherapy is increasingly used to treat ocular surface tumors as a primary therapy and an adjuvant treatment after surgical excision. The most employed topical agents include mitomycin C (MMC), 5-fluorouracil (5-FU), and interferon alpha-2b (IFNα2b), each with distinct mechanisms of action, efficacy profiles, and toxicity risks. Although these agents offer effective tumor control and allow for a non-invasive approach in many cases, ocular surface complications requiring medical or surgical management can occur. This summarizes the adverse effect and outilines practical strategies for their prevention and treatment. MMC is the most potent agent but also the most toxic, with reported complications such as limbal stem cell deficiency, punctal stenosis, and persistent epithelial defects. 5-FU demonstrates a more favorable safety profile, although rare cases of corneal ulceration have been described. IFNα2b is well tolerated and associated primarily with mild, reversible reactions. The choice of the proper agent should be tailored according to patient’s clinical presentation, ocular surface status, and ability to adhere to therapy and follow-up. Timely recognition and management of complications are essential to minimize long-term sequelae. Reliance on compounded formulations highlights the need for stable, standardized, and commercially available topical agents specifically designed for ocular use to ensure safety, reproducibility, and global accessibility.

## 1 Introduction

**Figure 1:**
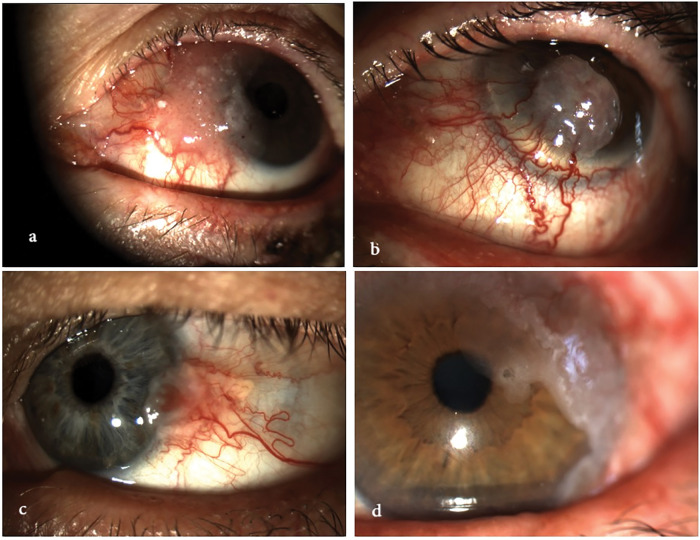

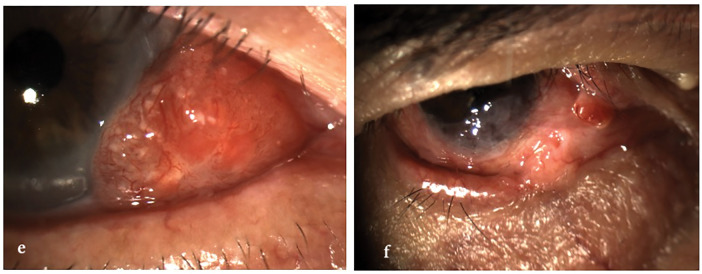
Clinical spectrum of ocular surface squamous neoplasia (OSSN). The image illustrates the morphological variability of OSSN, ranging from mild conjunctival intraepithelial neoplasia to fully invasive squamous cell carcinoma. Lesions may appear as gelatinous, leukoplakic, papilliform, or nodular, and can affect both the conjunctiva and cornea, with or without limbal involvement. All images were acquired using the SLITLAMP MICROSCOPE 700GL (Takagi Seiko Co., Ltd., Japan) integrated with the EyeGest Push.Print TD-10 software (version 6.0.10; Frastema Ophthalmics S.r.l., Varese, Italy) during routine clinical practice. **(a)** Early conjunctival intraepithelial neoplasia with mild epithelial thickening and minimal vascular changes. **(b)** Nodular, opaque lesion with feeder vessels, consistent with invasive squamous cell carcinoma. **(c)** Papilliform lesion involving the bulbar conjunctiva, suggestive of moderate-grade OSSN. **(d)** Leukoplakic lesion with limbal and corneal involvement, indicative of high-grade dysplasia. **(e)** Gelatinous, vascularized lesion of the inferior conjunctiva, consistent with low-grade OSSN. **(f)** Mixed nodular and gelatinous lesion with diffuse conjunctival and limbal involvement, suspicious for invasive carcinoma

Ocular surface tumors comprise a heterogeneous group of benign, premalignant, and malignant lesions affecting conjunctiva, cornea, and limbal epithelium [1]. Among malignant forms, the most common entities include squamous neoplasms, collectively referred to as ocular surface squamous neoplasia (OSSN), conjunctival melanoma, and conjunctival lymphomas. OSSN encompasses a wide spectrum of epithelial abnormalities, ranging from atypical hyperplasia and dyskeratosis to conjunctival intraepithelial neoplasia (CIN), and eventually to invasive squamous cell carcinoma (Fig. 1) [2–4]. Clinically, OSSN typically arises at the limbus, with variable extension onto the cornea or bulbar conjunctiva, sometimes mimicking benign conditions such as pterygium [5,6]. Rare presentations confined to the central cornea without limbal involvement have also been reported, posing diagnostic challenges [7]. OSSN may occasionally present with subtle or atypical features, mimicking chronic epithelial keratitis or medication-induced toxicity. In such cases, High-resolution optical coherence tomography (HR-OCT) can be instrumental in revealing a thickened, hyper-reflective epithelium with an abrupt transition zone, characteristic of epithelial dysplasia. Watane et al. reported a case of OSSN masquerading as recalcitrant keratitis, initially misdiagnosed as drug toxicity and ultimately confirmed by biopsy after imaging [8]. Similarly, Cechim et al. described a case of masquerade syndrome in OSSN, further supporting the need for early histologic confirmation in atypical presentations [9]. Early clinical suspicion and prompt biopsy remain essential to avoid delays in diagnosis, especially in lesions that mimic benign or inflammatory conditions. According to recent classifications, OSSN staging is based on American Joint Committee on Cancer Tumor-Node-Metastasis (AJCC TNM) criteria, which consider lesion size, depth, and limbal or scleral involvement. Such classification is essential to guide treatment selection and monitor prognosis [10]. The AJCC 8th edition classifies conjunctival squamous neoplasia using TNM criteria. Tis (tumor *in situ*) refers to confined intraepithelial lesions, while T1–T4 indicate increasing size and depth of invasion. T2 includes tumors invading the cornea or tarsal conjunctiva, T3 involves adjacent ocular structures such as sclera or eyelid, and T4 denotes orbital or beyond-orbit extension. Nodal (N) and metastatic (M) staging follow standard patterns. Accurate staging helps predict prognosis and guide management, especially in advanced or recurrent cases [10].

OSSN represents the most frequent malignant neoplasm of the ocular surface, with a reported global incidence ranging from 0.03 to 1.9 cases per 100,000 individuals per year that markedly increases in regions close to the equator [11,12].

**Figure 2:**
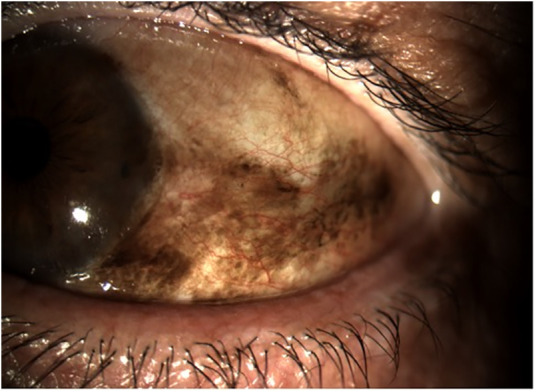
Clinical appearance of primary acquired melanosis (PAM) with cytological atypia. The lesion presents as flat, irregular brown pigmentation involving the bulbar conjunctiva and limbus. PAM with atypia is considered a precursor to conjunctival melanoma and requires close clinical monitoring and, in selected cases, surgical excision or adjunctive therapy. All images were acquired using the SLITLAMP MICROSCOPE 700GL (Takagi Seiko Co., Ltd., Japan) integrated with the EyeGest Push.Print TD-10 software (version 6.0.10; Frastema Ophthalmics S.r.l., Varese, Italy) during routine clinical practice

Conjunctival lymphoma, particularly of the mucosa-associated lymphoid tissue (MALT) subtype, is another relevant conjunctival malignancy, especially in Asian populations where it surpasses in incidence both OSSN and melanoma [13]. Conjunctival melanoma, though rare, carries a potentially serious prognosis, particularly when it arises from primary acquired melanosis (PAM) with cytological atypia [2,14]. A representative case of PAM with cytological atypia is shown in Fig. 2, highlighting its malignant potential.

In the case of OSSN, the most thoroughly documented risk factor is ultraviolet B (UVB) exposure, contributing to p53 gene mutations and direct damage to limbal stem cells [15]. Additional predisposing factors include infection with human papillomavirus (HPV) or human immunodeficiency virus (HIV), vitamin A deficiency, cigarette smoking, oculocutaneous albinism, and both iatrogenic and post-transplant immunosuppression [14,16]. In HIV-positive individuals, OSSN often presents at a younger age, with more aggressive clinical features and a higher likelihood of recurrence [17,18]. A retrospective analysis of over 200 OSSN lesions treated with interferon alpha-2b (IFN-α2b) showed that patients with a history of smoking more frequently presented with bilateral disease, dome-like tumor morphology, and greater involvement of the inferior conjunctiva. While initial tumor control was comparable between smokers and nonsmokers, the rate of recurrence was significantly higher among smokers, suggesting that tobacco exposure may negatively influence long-term treatment outcomes with interferon-based therapy [19]. Other surface malignancies such as melanoma and lymphoma have distinct risk profiles, primarily related to genetic factors, pre-existing lesions (e.g., PAM), and immune status.

In recent years, increased awareness and earlier diagnosis of ocular surface tumors have led to the development of more conservative treatment approaches [20]. In particular, the introduction and growing use of topical chemotherapy and immunomodulation have expanded the therapeutic armamentarium, offering an effective adjunct or alternative to surgery in selected cases [21]. This paradigm shift has raised new issues about efficacy, safety, and optimal indications for topical therapy in the management of these tumors.

Among conservative treatment options, topical therapy has emerged as a cornerstone in the management of OSSN [22]. Agents such as mitomycin C (MMC), 5-fluorouracil (5-FU), and IFN-α2b have demonstrated strong efficacy and an acceptable safety profile in both *in situ* and microinvasive diseases, offering a non-surgical alternative in selected cases or serving as adjuvant treatment after surgery when lesion margins are positive [23–26].

In contrast, topical therapy does not play an established clinical role in the management of the other two major malignant ocular surface tumors—conjunctival melanoma and conjunctival lymphoma. In conjunctival melanoma, MMC has been explored experimentally in cases of PAM with atypia, aiming at preventing the malignant transformation. However, for invasive melanoma, there is currently no indication for topical treatment and the standard of care remains complete surgical excision, typically combined with cryotherapy and, in selected cases, supplemented by radiotherapy or systemic immunotherapy [14]. Conjunctival lymphoma originates from subepithelial lymphoid tissue and is not responsive to topical agents. Management is generally based on low-dose radiotherapy for localized disease, while more aggressive or disseminated subtypes may require systemic therapies, including anti-CD20 monoclonal antibodies [2]. The aim of this review is to provide clinicians with a comprehensive, practical overview of adverse effects associated with topical chemotherapy for OSSN, and to outline evidence-based management strategies to minimize ocular surface complications.

## 2 Methods

A comprehensive literature search was conducted using the PubMed database to identify relevant studies on topical chemotherapy for ocular surface tumors, with a particular focus on adverse effects and iatrogenic complications. The search strategy combined terms related to ocular surface neoplasms and topical treatment, including the following keywords: “ocular surface” OR “conjunctival” OR “corneal” AND “tumors” OR “squamous” OR “neoplasia” AND “topical treatment” OR “mitomycin C” OR “5-fluorouracil” OR “interferon alpha” OR “photodynamic therapy” AND “toxicity” OR “iatrogenic” OR “complication” OR “safety”.

The search was limited to studies published in English language between 1995 and March 2025. A total of 59 full-text articles were initially retrieved. After screening titles and abstracts, 29 articles were excluded due to irrelevance to the search objectives, inconsistency with the study topic, or classification as individual case reports. The final review included 30 studies that met the inclusion criteria and provided data on the efficacy, safety, and clinical management of topical therapies for ocular surface tumors, with specific emphasis on MMC, 5-FU, IFNα2b. Following the initial screening, additional case reports and observational studies were included post hoc due to their relevance in illustrating rare but clinically significant adverse effects associated with topical therapy. The final review included 71 references encompassing retrospective series, prospective trials, meta-analyses, and selected case reports deemed essential for a comprehensive assessment of efficacy and safety profiles.

## 3 Topical Chemotherapeutic Agents for OSSN

The primary agents used in the topical treatment of OSSN are MMC, 5-FU, and IFN-α2b. These drugs differ in their mechanisms of action, clinical indications, and therapeutic profiles.

MMC is an antineoplastic antibiotic with alkylating properties that disrupts DNA replication by forming cross-links under anaerobic conditions and generating free radicals in aerobic environments [27]. MMC induces DNA alkylation and cross-linking by forming covalent bonds with guanine and cytosine residues, thereby preventing DNA replication and transcription. Its cytotoxicity is enhanced under hypoxic conditions, making it particularly effective in poorly vascularized neoplastic tissues [10]. It is non–cell-cycle specific and particularly effective in rapidly proliferating epithelial tissues. Its use is well established in ophthalmology for the management of superficial epithelial neoplasms, typically at concentrations ranging from 0.02% to 0.04%. The most frequently adopted dosing regimen consists of 4 daily applications (quater in die, QID), administered in one-week cycles alternating with one week off, usually for 2 to 4 cycles depending on clinical response and tolerability. MMC is among the most potent topical agents for OSSN and is especially indicated in aggressive or recurrent disease. In a randomized controlled trial, Hirst reported a complete clinical response in 93% of patients treated with topical 0.04% MMC compared to the absence of response in the placebo group [28]. However, its high cytotoxic potential and delayed-onset toxicity require careful case selection and close follow-up, as further discussed in Section 3 [29–32].

5-FU is a pyrimidine antimetabolite that inhibits thymidylate synthase, thereby interfering with DNA and RNA synthesis during the S-phase of the cell cycle [33]. It is typically used as a 1% ophthalmic solution, administered QID for one week, followed by a 3-week drug holiday, in repeated cycles. Most treatment protocols recommend 2 to 4 cycles, although this may vary depending on lesion size and clinical response. 5-FU is widely available, inexpensive, and does not require refrigeration, making it a practical option for outpatient or resource-limited settings. It has shown good efficacy in early to intermediate disease and is generally well tolerated, with most side effects being mild and self-limiting [23,25,34]. In a prospective case series by Rudkin and Muecke, 5-FU was used as an adjuvant following surgical excision, achieving a recurrence rate of only 1.5% over at least 12 months of follow-up, even in cases with positive surgical margins [35].

IFN-α2b is a recombinant cytokine with immunomodulatory, antiproliferative, and antiviral properties [36]. Its antitumor effects are mediated by enhanced immune surveillance, inhibition of angiogenesis, and induction of apoptosis. The drug also exerts antiangiogenic and antiproliferative activity while sparing healthy conjunctival and limbal cells, which accounts for its excellent tolerability profile [10]. IFN-α2b may be administered either topically, at a concentration of 1 million IU/mL QID, or via subconjunctival injection at doses ranging from 3 to 10 million IU once weekly [24,37–39]. Topical therapy is typically continued until complete clinical resolution is achieved, which may require several weeks to months. IFNα2b is extremely well tolerated, even during prolonged treatment, and is particularly suited for patients with pre-existing ocular surface disease or for those requiring a more conservative approach [40,41]. In a prospective study by Sturges et al., IFN-α2b induced complete tumor regression in all treated patients, with no recurrences observed over a median follow-up of 35.6 months [42]. Similarly, Shields et al. reported complete response rates ranging from 75% to 100% depending on tumor stage, with a recurrence rate of 5% in patients treated with topical IFN-α2b alone [43]. While its onset of action is slower and access may be limited by the need for compounding and cold storage, it remains a preferred option when long-term safety is a priority [44]. Emerging evidence also supports its off-label use in cases of primary acquired melanosis with atypia and selected conjunctival melanomas [45,46].

In summary, MMC, 5-FU, and IFNα2b offer complementary therapeutic options that support tailored treatment strategies based on tumor severity, ocular surface status, and logistical factors such as drug availability and patient compliance. A 2023 review by Monroy et al. analyzed 35 studies encompassing 1571 eyes treated with topical agents for OSSN, reporting complete resolution rates of 81%–100% with IFNα2b, 82%–100% with 5-FU, and 79%–100% with MMC. Recurrence rates ranged from 0%–20% for IFNα2b, 0%–11% for 5-FU, and 0%–15.1% for MMC. These findings confirm the high efficacy of all three agents, while suggesting a slightly lower recurrence risk with IFNα2b in certain subgroups. Nonetheless, variability in patient selection, lesion stage, and follow-up duration across studies limits direct comparisons. These data further support the need for individualized treatment planning based on tumor size, location, prior therapies, and ocular surface integrity [47]. A comparative overview is provided in Table 1, while practical recommendations for managing treatment-related complications are presented in Section 3.

**Table 1:** Comparative summary of topical chemotherapeutic agents for OSSN, including efficacy, adverse events, and cost considerations

Parameter	Mitomycin C (MMC)	5-fluorouracil (5-FU)	Interferon alpha-2b (IFN**α**2b)
**Complete resolution rate**	80%–100% [30–32]	80%–90% [39,48–50]	80%–90% [37,38,40,44]
**Average response time**	4–6 weeks [31,32]	4–8 weeks [48,49]	8–16 weeks [40,44]
**Common adverse events**	Punctate keratopathy, punctal stenosis, LSCD^1^ [2,30–32]	Hyperemia, punctate keratitis, lid erythema [15,39,48,50,51]	Hyperemia, follicular conjunctivitis, flu-like symptoms [37,38,40,44]
**Severe adverse events**	High (up to 80%) [30–32]	Moderate (10%–30%) [15,48,49,51]	Low (<10%) [37,40,44]
**Treatment discontinuation**	5%–18% [30,32]	Rare [48,49,51]	Rare [37,44]
Availability*	Commercially available [31]	Widely available [48]	Requires compounding in many settings [37,44,52]
Cost per treatment cycle*	Low (~€8–9) [48,52]	Low (€165.57 ± 45.85) [53]	High (€1025 ± 130.68) [53]; also reported ~$100–300 [44]
**Ideal clinical setting**	Extensive/recurrent lesions [30–32]	Intermediate lesions, cost-sensitive settings [48–50]	Initial or fragile surface disease [38,40,44,45]
**Relative contraindications**	Dry eye, pre-existing LSCD [31,32]	Ocular hypersensitivity [15,51]	Access, cost, slower onset [44,52]

Note: *Costs and availability are approximate and based on published studies and clinical estimates; ^1^LSCD, limbal stem cell deficiency.

In clinical practice, some patients may receive sequential or combined topical chemotherapy regimens when response to a single agent is inadequate or contraindicated. Although high-level comparative evidence is limited, several studies have described the use of MMC followed by IFNα2b in cases with partial or slow response, or the addition of 5-FU after initial treatment failure [54]. Rare presentations of OSSN confined to the central cornea without limbal involvement have also been described, requiring combined strategies such as surgical excision with intraoperative MMC, followed by postoperative topical 5-FU to ensure complete resolution and prevent recurrence [7]. In their review, Monroy et al. reported that sequential therapy, particularly MMC followed by IFNα2b or *vice versa*, is frequently used in clinical settings to improve response in extensive or resistant OSSN [47]. In selected cases, topical agents may be employed after surgical excision and cryotherapy to treat residual or subclinical disease, a strategy known as adjuvant chemoreduction [55]. Conversely, preoperative topical treatment may be used to reduce tumor size before surgical excision. Nonetheless, in locally advanced or invasive cases (T2+), monotherapy is often insufficient. Combined approaches involving surgery followed by adjuvant high-dose-rate interventional radiotherapy (HDR-IRT) have been explored. However, recent data show limited success in OSSN, with recurrence rates up to 80%, underscoring the need for tailored protocols and better local control strategies in aggressive subtypes [56]. These multimodal approaches may influence both efficacy and toxicity and should be tailored to the patient’s condition, with close monitoring for cumulative adverse effects. Further studies are needed to standardize these protocols.

Access to topical chemotherapy agents varies significantly across geographic regions, reflecting broader disparities in ocular oncology care. While interferon alfa-2b is no longer commercially available in the United States due to market withdrawal by Merck, it remains accessible in parts of Europe and through compounding pharmacies. Conversely, the availability of mitomycin C and 5-FU depends on national regulatory policies and healthcare infrastructure, with notable challenges reported in low- and middle-income countries. These disparities are part of a broader pattern of unequal access to ocular oncology treatments, particularly affecting Black and socioeconomically disadvantaged patients with OSSN in both high- and low-income settings [12].

## 4 Adverse Effects and Their Management

While topical chemotherapy is an effective, minimally invasive treatment for OSSN, it can be associated with a range of ocular surface complications [57]. The type and severity of adverse effects vary depending on the agent used and the baseline condition of the ocular surface. Among the commonly used agents, MMC carries the highest risk of toxicity, followed by 5-FU, while IFNα2b is generally well tolerated and has the most favorable safety profile.

### 4.1 Mitomycin C

MMC is the most potent and toxic among the topical agents used for OSSN. Adverse effects may be early or delayed and range from mild discomfort to potentially vision-threatening complications. Common findings include conjunctival hyperemia, foreign body sensation, and superficial punctate keratitis. In more severe cases—particularly in eyes with compromised ocular surfaces or prolonged exposure—patients may develop persistent epithelial defects, punctal stenosis, chronic epiphora, and limbal stem cell deficiency (LSCD) [58,59]. These complications are more frequent in cases with extensive limbal involvement or pre-existing surface fragility [4]. Although specific data on MMC concentrations in OSSN are limited, evidence from glaucoma surgery supports the use of lower concentrations to mitigate risk. In a mega-analysis comparing MMC 0.2 and 0.4 mg/mL during polystyrene-isobutylene-styrene microshunt implantation, the higher concentration was associated with significantly better outcomes without a corresponding increase in serious adverse events [60]. Long-lasting cytological changes in the conjunctival epithelium have also been observed, which can persist for months after treatment and may complicate clinical monitoring [30,31]. Management should be guided by the severity of symptoms. Mild toxicity often resolves with temporary suspension of MMC and supportive therapy, including preservative-free tear substitutes and low-dose topical corticosteroids (e.g., fluorometholone or loteprednol). When punctal stenosis causes symptomatic epiphora, silicone punctal plugs may be used. If symptoms persist, surgical punctoplasty can be considered. Partial LSCD may require more targeted interventions. In the acute phase, transplantation of amniotic membrane can help restore the epithelial surface and reduce inflammation. If epithelial recovery fails, limbal stem cell transplantation (such as SLET—Simple Limbal Epithelial Transplantation) from the contralateral eye may be indicated. In cases of bilateral involvement, allogeneic transplantation from a living or cadaveric donor may be necessary [40,45]. If corneal ulceration or stromal melting develops, MMC must be stopped immediately. Corneal protection strategies may include bandage contact lenses, autologous serum eye drops or plasma rich in growth factors, and, in more severe cases, amniotic membrane transplantation. Eyes at risk of perforation may benefit from temporary tarsorrhaphy or fibrin glue application [29,32].

### 4.2 5-Fluorouracil

5-FU is generally better tolerated than MMC [61]. Most patients experience only mild and transient adverse events, such as redness, ocular irritation, punctate keratitis, or lid skin inflammation [62]. In rare cases, more severe complications like corneal epithelial breakdown or stromal melting have been reported, especially in eyes with pre-existing ocular surface disease [58,63]. Although uncommon, corneal endothelial toxicity has also been reported with prolonged topical use of 5-FU. In a recent case, bilateral endothelial decompensation with pigmentary dusting and guttata was observed after multiple treatment cycles, documented by specular microscopy. The changes resolved after discontinuation, suggesting a possible dose-related effect in susceptible individuals [64].

**Figure 3:**
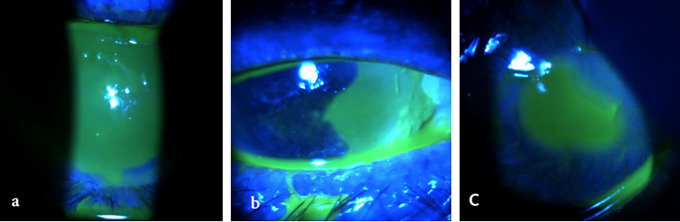
Corneal complications related to topical 5-fluorouracil therapy. All images were acquired using the SLITLAMP MICROSCOPE 700GL (Takagi Seiko Co., Ltd., Japan) integrated with the EyeGest Push.Print TD-10 software (version 6.0.10; Frastema Ophthalmics S.r.l., Varese, Italy) during routine clinical practice. **(a)** Delayed epithelial defect after treatment suspension in a patient with paralytic ectropion. **(b)** Epithelial defect induced by treatment, necessitating temporary discontinuation for complete resolution. **(c)** Severe corneal ulceration with impending perforation in a patient with extreme tear deficiency following dacryoadenectomy

Management of these adverse events typically involves the interruption of treatment and the initiation of frequent lubrication with preservative-free tear substitute. In cases characterized by inflammation, a short course of topical corticosteroids can be started. If epithelial damage is significant, additional measures such as bandage lenses or autologous serum drops may be necessary [65]. Treatment can often be resumed once symptoms have resolved, depending on the clinical scenario [15,39,49,50]. Clinical examples of corneal complications related to 5-fluorouracil therapy are given in Fig. 3.

### 4.3 Interferon Alpha-2b

Among the topical agents used for OSSN, IFNα2b has the most favorable safety profile. Adverse effects are rare, mild, and self-limiting. These include conjunctival hyperemia, mild follicular reaction, and, in the case of subconjunctival injections, transient flu-like symptoms. No cases of serious or long-term ocular toxicity have been reported [58].

In most situations, treatment can be continued without interruption. Mild irritation is usually manageable with a tear substitute or occasional use of low-potency corticosteroids. For patients receiving subconjunctival injections, flu-like symptoms can be treated with paracetamol if needed. Overall, IFNα2b is well tolerated even during extended treatment periods, and discontinuation is rarely necessary [24,38,44,45,48]. Although IFNα2b is generally well tolerated, recent case reports have described rare immune-mediated ocular complications. One such case involved presumed reactivation of herpes simplex virus (HSV)-associated endothelial keratitis in a patient without prior herpetic history during treatment for OSSN [66]. Another report documented the development of acute fibrinous anterior uveitis in a patient with a known susceptibility associated with the human leukocyte antigen B27 (HLA-B27), which is commonly linked to autoimmune ocular inflammation [67]. In that case, inflammation resolved with corticosteroid treatment and IFNα2b was safely resumed. These findings suggest a potential immunomodulatory effect of IFNα2b and warrant caution in individuals with a known history or predisposition to autoimmune ocular inflammation.

In a recent meta-analysis including over 500 treated eyes, the most frequent adverse event across all treatment modalities was dry eye, particularly after surgical excision (59%). Hyperemia occurred in 44% of cases treated with MMC, while keratopathy was reported in approximately 12%. In contrast, IFNα2b demonstrated a more favourable safety profile, with only 12.9% of patients reporting systemic flu-like symptoms [68]. A synthesis of common ocular surface complications and their frequencies, drawn from multi-study data including meta-analyses and long-term case series, is presented in Table 2. These data may assist clinicians in anticipating and managing adverse effects during topical chemotherapy for OSSN.

**Table 2:** Progression and management of adverse effects in topical OSSN therapy

Clinical scenario	Suggested management	May progress to	Reported frequency **(%)**
**Redness, irritation, SPK**	Suspend drug; preservative-free lubricants; ±mild topical corticosteroids	Persistent symptoms, LSCD	MMC: 44%, 5-FU: 43%, IFN: 14% [68]
**Persistent symptoms, epiphora**	Assess for punctal stenosis or LSCD; treat accordingly	Punctal stenosis, LSCD	MMC: 36%, 5-FU: 22% [68]
**Punctal stenosis**	Temporary punctal plugs; surgical punctoplasty if needed	Chronic tearing, ocular surface instability	MMC: 4%–8%, 5-FU: 5.6% [48,51]
**Partial LSCD**	Amniotic membrane; consider SLET; allogeneic transplant in bilateral cases	Full LSCD with epithelial breakdown	5-FU: 3% (long-term cases) [39]
**Corneal ulceration or stromal melting**	Urgent discontinuation: bandage lens, amniotic membrane, autologous serum; tarsorrhaphy	Perforation, permanent vision loss	MMC: 1%–3%, 5-FU: rare (<1%) [31]
**Flu-like symptoms (IFNα2b systemic)**	Continue treatment; symptomatic relief (e.g., paracetamol)	Rarely progresses	IFNα2b: 12.9% [48]

Note: SPK, Superficial Punctate Keratitis; LSCD, Limbal Stem Cell Deficiency; SLET, Simple Limbal Epithelial Transplantation; MMC, mitomycin C; 5-FU, 5-fluorouracil; IFN, interferon; IFNα2b, interferon alpha-2b.

## 5 Discussion

Topical chemotherapy has become an integral part of the therapeutic armamentarium for OSSN, used both as a non-invasive alternative to surgical excision and adjuvant therapy following tumor resection [69]. One of the key advantages is the ability to treat the entire ocular surface, effectively targeting both clinically visible and subclinical lesions that may persist after surgery [31,32,40]. Among the available agents, MMC, 5-FU, and IFNα2b remain the most frequently used, each with unique pharmacological features, strengths, and limitations. These differences have been well documented in recent comparative reviews, which highlight IFNα2b as the agent with the most favourable safety profile, followed by 5-FU and MMC [47]. However, drug selection should remain flexible, taking into account lesion severity, ocular surface condition, drug accessibility, and the need for rapid tumor control. A 2022 meta-analysis by Kozma et al. found no significant differences in tumor resolution (Odds Ratio [OR]: 0.785; Confidence Interval [CI]: 0.130–4.736, *p* = 0.792) or recurrence (OR: 0.746; CI: 0.213–2.609, *p* = 0.646) between topical pharmacotherapy and surgical excision for OSSN. These results reinforce the role of medical management as a valid primary approach in selected patients [68]. Moreover, recurrence rates after surgical excision alone remain considerable, with 13% at one year, 31% at five years, and up to 56% at 15 years, particularly in the presence of positive margins or limbal involvement [4].

MMC is widely recognized for its strong cytotoxic efficacy, particularly in patients with extensive, recurrent, or aggressive disease. Multiple studies have shown complete resolution rates above 90% when used appropriately [30,32]. However, its potential for ocular surface toxicity is significant and includes punctate keratopathy, epithelial erosions, punctal stenosis, and, in severe cases, LSCD [29,31]. Long-term alterations in conjunctival cytology have also been described [30], underscoring the importance of close monitoring during and after therapy. MMC is best reserved for patients with robust ocular surfaces and for those who require rapid and decisive tumor control. Although both 0.02% and 0.04% concentrations of MMC are used in clinical practice, there is currently a lack of prospective comparative studies evaluating their differential impact on ocular surface toxicity in OSSN treatment. Future research should address whether higher MMC concentrations lead to increased efficacy without proportionally greater adverse effects, as has been suggested in surgical settings such as glaucoma microshunt implantation [60].

5-FU represents a middle ground in the balance between efficacy and tolerability. Its antimetabolite action is effective, particularly in early or moderate disease, and it has been widely used both as a primary and secondary therapy [40,49]. Its side effects are generally milder than those of MMC, with conjunctival irritation, hyperemia, and superficial keratitis being the most reported. Severe complications such as stromal melting are rare but documented [15,50]. The drug’s favorable cost profile and availability make it a valuable option in many clinical settings. Still, careful follow-up is essential, especially during treatment cycles, to detect early signs of toxicity.

IFNα2b distinguishes itself for its excellent safety profile. Its mechanism, immunomodulatory rather than directly cytotoxic, results in a minimal incidence of ocular surface damage, and most side effects are mild and self-limited [38,40]. These include follicular conjunctivitis, hyperemia, and, in cases of subconjunctival administration, transient flu-like symptoms. Its main disadvantages lie in its longer time to respond, the need for prolonged treatment (often up to several months), and the practical limitations associated with compounding, refrigeration, and inconsistent availability [37,44]. Nevertheless, its high tolerability makes it an excellent first-line choice for patients with delicate ocular surfaces or when long-term safety is a priority. The most recent and largest long-term cohort on IFNα2b monotherapy by Sripawadkul et al. showed a tumor resolution rate of 80.4% with a mean treatment duration of 4.8 months. The recurrence rate remained as low as 3.1% at 5 years, confirming the sustained efficacy of IFNα2b even in patients followed for over two decades [70]. Predictive factors for treatment response with IFNα2b include female sex and superior tumor location, while immunosuppressive conditions such as HIV or atopy were associated with non-resolution [70]. While topical administration is preferred due to its safety and convenience, subconjunctival injections may lead to faster resolution in select patients, although with increased flu-like symptoms and discomfort [10].

The selection of the optimal topical agent must be individualized. Factors such as lesion size and severity, pre-existing ocular surface disease, anticipated compliance, and logistical access to the medication should all be considered. As highlighted, careful follow-up is not only essential to detect adverse events but also to verify treatment response, ensure resolution, and monitor for recurrence. In locally advanced or invasive OSSN (≥T2), monotherapy with topical agents may be insufficient. Combined protocols involving surgical excision and adjuvant high-dose-rate interventional radiotherapy (HDR-IRT) have been explored, although recent data suggest limited efficacy in OSSN, with recurrence rates approaching 80% despite successful outcomes in eyelid tumors [56]. These findings underscore the need for tumor-specific strategies and improved local control protocols in aggressive ocular surface neoplasms. Additionally, the coexistence of OSSN with microbial keratitis (MK), although rare, has been identified as a significant negative prognostic factor. In a large cohort, Kapoor et al. reported this association in 2% of patients, frequently involving HIV-positive males with noduloulcerative, pigmented lesions and corneal or scleral extension. These cases were associated with low rates of vision and globe salvage despite aggressive treatment [71]. Even after apparent clinical remission, periodic monitoring remains recommended, as late recurrences have been described [39].

Despite encouraging results, the current body of evidence remains limited by methodological heterogeneity and lack of standardization. Most studies are retrospective, with variable treatment protocols and short follow-up. In addition, commercially standardized topical formulations are lacking, and most agents are still obtained through compounding pharmacies, contributing to variability in efficacy and safety across settings.

Looking ahead, the development of globally available, standardized formulations (stable at room temperature, with consistent pharmacodynamics and regulatory support) would represent a key advancement. Future efforts should also aim to integrate TNM staging more systematically into treatment protocols, especially in clinical trials, to enable better stratification of risk and outcomes. These innovations would improve drug accessibility, ensure reproducibility, and support equitable care delivery.

In summary, the choice among MMC, 5-FU, and IFNα2b should be guided by efficacy, toxicity profile, patient-specific features, and local availability. Rigorous follow-up is essential to ensure early detection of complications and long-term control of OSSN. Continued research, clinical harmonization, and pharmaceutical development remain priorities.

***Limitations of the Literature and Lack of Standardized Formulations***

While topical chemotherapy for OSSN has gained wider adoption, the literature remains limited by several methodological weaknesses. Most studies are retrospective, with small and heterogeneous cohorts, variable inclusion criteria, and inconsistent definitions of treatment success. Direct comparisons between agents are rare, and long-term follow-up beyond 12–24 months is uncommon.

Adverse events are often underreported or not stratified by dose, duration, or ocular surface condition, making cross-study comparisons difficult. Additionally, newer approaches such as combination or sequential topical therapy have been explored only in limited series, often lacking robust outcome data.

Well-designed prospective trials and multicenter registries are needed to address these limitations, define optimal treatment protocols, and identify patient-specific predictors of success and toxicity.

## 6 Conclusions

Topical chemotherapy remains a cornerstone in the management of OSSN, offering a non-invasive and effective alternative to surgery in selected cases. MMC, 5-FU, and IFNα2b differ in efficacy, toxicity, and availability, supporting a personalized approach based on clinical severity, surface integrity, and access to care. MMC is potent but carries greater toxicity; 5-FU offers a balanced profile; and IFNα2b remains the safest, particularly for fragile ocular surfaces, despite its logistical limitations.

Careful follow-up and early recognition of adverse effects are essential to ensure long-term control and avoid permanent sequelae. Sequential or combined regimens may improve response in resistant or recurrent disease, while multimodal strategies are under investigation for invasive cases. However, disparities in access and the lack of standardized commercial formulations still hinder global equity in care.

Moving forward, harmonized treatment protocols, stable drug formulations specifically developed for ophthalmic use, and broader availability will be critical to improving therapeutic consistency. The current evidence confirms that, when selected appropriately, topical agents can achieve long-term tumor control comparable to surgery, while preserving the ocular surface and minimizing morbidity.

## Data Availability

Not applicable.
